# Same label, different patients: Health-workers’ understanding of the label ‘critical illness’

**DOI:** 10.3389/frhs.2023.1105078

**Published:** 2023-02-07

**Authors:** Elibariki Mkumbo, Tamara Mulenga Willows, Onesmus Onyango, Karima Khalid, John Maiba, Carl Otto Schell, Jacquie Oliwa, Jacob McKnight, Tim Baker

**Affiliations:** ^1^ Department of Health Systems, Ifakara Health Institute, Dar es Salaam, Tanzania; ^2^ Health Systems Collaborative, Department of Tropical Medicine and Global Health University of Oxford Health, Oxford, United Kingdom; ^3^ Health Services Unit, KEMRI-Wellcome Trust Research Programme, Nairobi, Kenya; ^4^ Department of Anaesthesia, Muhimbili University of Health and Allied Sciences, Dar es Salaam, Tanzania; ^5^ Department of Global Public Health, Karolinska Institutet, Stockholm, Sweden; ^6^ Centre for Clinical Research Sörmland, Uppsala University, Eskilstuna, Sweden; ^7^ Department of Medicine, Nyköping Hospital, Nyköping, Sweden; ^8^ Department of Clinical Research, London School of Hygiene and Tropical Medicine, London, United Kingdom

**Keywords:** labelling, understanding, critical illness, critical care, emergency care, communication, healthcare workers, definition

## Abstract

**Background:**

During the course of patients’ sickness, some become critically ill, and identifying them is the first important step to be able to manage the illness. During the course of care provision, health workers sometimes use the term ‘critical illness’ as a label when referring to their patient's condition, and the label is then used as a basis for communication and care provision. Their understanding of this label will therefore have a profound impact on the identification and management of patients. This study aimed to determine how Kenyan and Tanzanian health workers understand the label ‘critical illness’.

**Methods:**

A total of 10 hospitals—five in Kenya and five in Tanzania—were visited. In-depth interviews were conducted with 30 nurses and physicians from different departments in the hospitals who had experience in providing care for sick patients. We conducted a thematic analysis of the translated and transcribed interviews, synthesized findings and developed an overarching set of themes which captured healthcare workers’ understandings of the label ‘critical illness’.

**Results:**

Overall, there does not appear to be a unified understanding of the label ‘critical illness’ among health workers. Health workers understand the label to refer to patients in four thematic ways: (1) those in a life-threatening state; (2) those with certain diagnoses; (3) those receiving care in certain locations; and (4) those in need of a certain level of care.

**Conclusion:**

There is a lack of a unified understanding about the label ‘critical illness’ among health workers in Tanzania and Kenya. This potentially hampers communication and the selection of patients for urgent life-saving care. A recently proposed definition, “*a state of ill health with vital organ dysfunction, a high risk of imminent death if care is not provided and the potential for reversibility*”, could be useful for improving communication and care.

## Introduction

During the course of an illness, some patients become critically ill. Critical illness is common, with an estimated 45 million adults becoming critically ill globally every year (1). An important function of health care facilities is the ability to identify which patients, among all the others, are critically ill in order to manage them quickly and appropriately (2, 3). Indeed, providing quality care to critically ill patients has the potential to reduce mortality (4).

‘Critical illness’ can be regarded as a *label* that health workers use during the course of care provision. Social labelling theory describes how human beings tend to group phenomena together based on certain features or characteristics—these groups are referred to as ‘labels’ (5, 6). Labels in healthcare are seen as useful as they provide a framework from which to organize and interpret clinical symptom presentations, support clinical decision making through directing treatment decisions, and provide information on possible condition course and overall prognosis (7, 8). These labels also allow healthcare workers to assume homogeneity within groups and frame experience and knowledge in addition to providing an efficient method for health workers to communicate (9). Health workers may use labels to describe particular patients due to diagnostic results, or they may use them for descriptive features in patients (10).

While labels are certainly useful, they can be understood differently by different people, which has the potential to cause miscommunication and ambiguity (11). The same label may be used by one health worker for a patient with a particular symptom, by another for a patient in a particular situation, and by a third for a patient receiving a particular therapy (12, 13). When different understandings underlie the same label, communication can be impaired, impacting the quality of care (14).

A health workers’ understanding of the label ‘critical illness’ is a key aspect in their identification and management of these patients, but to our knowledge, there has not been any work looking at such understandings. This study therefore aimed to describe how Kenyan and Tanzanians health workers understand the label ‘critical illness’.

## Material and methods

### Study design

We used an exploratory, qualitative approach, as recommend for less studied subjects (15). We used in-depth interviews as described by Braun (16) to determine health workers understanding of the label ‘critical illness’. A thematic analysis allowed us to study the perspectives of the research participants, and identify similarities and differences (17).

### Setting

This study was part of the project “Provision of Essential Treatment in Critical Illness” (POETIC) investigating the care of critically ill patients in Kenya and Tanzania. The study was conducted in five Kenyan and five Tanzanian hospitals. Kenya and Tanzania are lower middle-income countries in East Africa. In both countries, the included hospitals were government-owned at primary, secondary and tertiary levels. They were purposefully selected to represent various levels of care and relative ease to access due to challenges around travel in the COVID-19 pandemic.

### Participants and recruitments

A purposive sampling method (18) was used to identify participants in the hospitals. Potential participants were nurses and physicians from intensive care units, emergency units, out-patient departments, pediatric departments, medical wards, maternity wards and labor wards who had experience in providing care for sick patients. We contacted the hospital's adminstration, explained our study and requested information about eligible participants, considering diversity on work experience, department and cadre. Eligible participants were given an explanation regarding the purpose of the study, the procedure, privacy and confidentiality, and their right to end the interview at their convenience. Participants provided written consent for their participation and for the recording of the interview. The number of participants to interview was guided by thematic saturation in which additional data no longer led to any new emergent themes’ (19), and was expected to include 15–20 in each country.

### Ethical considerations

This study was granted ethical approval by the Kenya Medical Research Institute Scientific and Ethics Review Board (SERU Number 4085), Tanzanian National Institute for Medical Research (NIMR/HQ/R.8a/Vol.IX/3537) and London School of Hygiene and Tropical Medicine (REF 22 866). All participants were assured that audio recordings of interviews would be deleted once interviews were transcribed and gave consent for the anonymous use of quotes from interviews.

### Data collection

Interviews took place between January and December 2021 during the COVID-19 pandemic. Researchers with qualitative research experience (EM, JM, KK and OO) conducted the interviews which lasted between 45 and 60 min. Interviews in Tanzania were done physically and were conducted in Swahili, the national language, and in Kenya were done virtually in English due to COVID-19 travel restrictions.

Interviews used questions and subsequent probing depending on the information provided by the participant to explore understandings of the label ‘critical illness’. Example questions included: (i) What is critical illness? (ii) Which patients are critically ill? (iii) Where are critically ill patients in the hospital?

### Data processing

All audio recorded interviews were transcribed verbatim by a person fluent in the language and the Swahili was then translated into English. Translated transcripts were cross-checked by the experienced qualitative researcher EM, by comparing interview notes, translations and completeness before data were coded. Data validation was done by the researchers in the form of data reflection meetings of key findings with the facilities and any discrepancies noticed were discussed with facilities and adjustments made.

### Data analysis

The transcripts were uploaded to NVivo-12 qualitative data software and read several times to gain familiarization with the data. To explore health workers understanding of the label, a recommended content analysis approach was used (20), where data were thematically coded, analyzed and interpreted using inductive analysis (21). Content that specifically contained participants’ reflections on the meaning, understanding, description or classification of critically ill patients was organized into codes (22). The codes were organized into categories and themes which were iteratively revised as new codes emerged through the process. This was followed by data verification focused on validity where two social scientists (EM, JM) separately coded a few transcripts and then compared themes found, discussed discrepancies and set standards to guide the whole coding work. Transcripts and codes were rechecked allowing the researcher to verify or modify hypotheses already arrived at previously. The final stage involved a clinical expert (TB) who cross-checked the validity and applicability of the identified themes in the clinical setting. Themes developed were refined and interpreted to reflect health workers’ understanding of the label ‘critical illness’.

## Results

A total of 30 health workers—12 doctors and 18 nurses—in the 10 study hospitals were interviewed (Table 1).

**Table 1:** Characteristics of participants.

Country	Hospital name	Hospital level	Cadre	Gender	Department
Kenya	Hospital 1	Level 6 (tertiary)	Nurse	Male	Emergency Department
			Nurse in-charge	Female	Intensive Care Unit
			Nurse	Male	Medical Ward
			Nurse	Male	Labor Ward
	Hospital 2	Level 4 (secondary)	Doctor	Male	Pediatric Outpatient
			Doctor	Male	Maternity Ward
			Nursing services manager	Female	Medical Ward
	Hospital 3	Level 5 (secondary)	Clinical officer (non-physician clinician)	Male	Out-patient Department
			Doctor	Female	Intensive Care Unit
			Nursing services manager	Male	Emergency Department
	Hospital 4	Level 5 (secondary)	Doctor	Male	Emergency Department
			Doctor	Female	Emergency Department
	Hospital 5	Level 4 (secondary)	Clinical Officer (non-physician clinician)	Female	Emergency/Out-patient Department
			Doctor	Male	Emergency/Out-patient Department
			Nurse	Female	Isolation Unit
Tanzania	Hospital 1	Regional referral	Emergency Medicine Specialist	Male	Emergency Department
			Internal Medicine Specialist	Female	Intensive Care Unit
			Nurse	Male	Emergency Department
	Hospital 2	District hospital	Doctor	Male	Out-patient Department
			Nurse	Female	Maternity Ward
			Nurse	Female	Medical Ward
	Hospital 3	Regional referral	Doctor	Male	Emergency Department
			Nurse	Female	Out-patient Department
			Nurse	Female	Surgical/Medical ward
	Hospital 4	National referral	Nurse	Female	Intensive Care Unit
			Nurse	Male	Emergency Department
			Nurse	Female	Pediatric Ward
	Hospital 5	District hospital	Doctor	Male	Medical Ward
			Doctor	Female	Maternity Ward
			Nurse	Male	Outpatient Department

Overall, there did not appear to be a unified understanding of the label ‘critical illness’ among healthcare workers. Health workers understood the label to refer to patients in four ways, as captured by the themes: (1) Those in a life-threatening state; (2) Those with certain diagnoses; (3) Those receiving care in certain locations; and (4) Those in need of a certain level of care.

### Theme 1: Those in a life-threatening state

Health workers described their use of the label ‘critical illness’ to refer to patients who are in a state which is life-threatening, meaning that if they were left untreated, it likely could result in death. Within the theme of life-threatening state, participants mentioned phenomena such as severe bleeding, difficulty in breathing, and inability to talk as examples that characterized a critically ill patient.

One nurse said:

*“By experience just by looking at them sometimes a person can have difficulty breathing, he/she cannot talk, you just see that the person is critically ill”*. (Nurse, Tanzania)

Some referred to a decreased level of consciousness as reflecting critical illness. This nurse pointed out that if the level of consciousness is low, to her that means a patient is critically ill.

*“Critically ill patient is the one whose level of consciousness has been decreased that is critically ill patient, level of consciousness has been decreased below normal”* (Nurse, Tanzania)

Some without mentioning a definitive life threatening condition, referred instead to the unresponsiveness of a patient as reflecting critical illness.

*“Critically ill patient, a patient who comes unresponsive, they really need assistance in, they are, some are not even talking, generally unresponsive patient, either it could be through accidents through a medical condition, anything like that”*. (Doctor, Kenya)

### Theme 2: Those with certain diagnoses

To some health workers, patients who had been diagnosed with certain conditions indicated that they were critically ill. Diagnoses such as eclampsia, head injury and cerebral malaria indicated critical illness, whereas diagnoses such as upper respiratory tract infection, diabetes and hypertension indicated that the patient was not critically ill.

*“She might have what we call pregnancy induced hypertension or eclampsia and she can develop eclampsia, meaning that she was ok but in between during labor she will develop such a condition, then she will become critical”*. (Nurse, Tanzania)

Severity of critical illness is also measured relative to how complicated a diagnosis is:

*“Like in maternity we've been involved in the care of some critically ill patients may be due to preeclampsia or eclampsia or maybe severe anaemia or maybe postpartum hemorrhage”* (Nurse, Tanzania)

### Theme 3: Those receiving care in certain locations

In hospitals, different departments such as pediatric, maternity, emergency and other specialized units such as Intensive Care Units (ICUs) and operating theatre have particular patients that they care for. Health workers associated critical illness with patients being treated in particular areas. To this health worker, being taken care of in the casualty department meant being critically ill:

*“The critically ill patients are seen in casualty, immediately they come, they’re triaged then they’re taken to casualty in our hospital, then they’re transferred to various departments or they’re referred depending with the condition of the patient”*. (Nurse, Kenya)

This also reflected in participants’ beliefs that there must be a specialized unit for handling critical illness and in its absence a ward has to be used:

*“In our hospital, we don’t have critical care unit, they are just taken to general ward”* (Clinical officer, Kenya)

### Theme 4: Those in need of a certain level of care

Some health workers suggested that they can tell a patient is critically ill by observing that a patient requires an upgrade to the level of care they are receiving. For example, a patient may suffer a complication requiring surgery, or they may develop breathing difficulties and need more advanced respiratory support.

This health worker mentioned a requirement for more medical support and oxygen as indicating critical illness:

*“A critically ill patient is a patient who needs advanced care this including the support, the medical support that including oxygen, feeding and needs definitive management, this patient needs to be on watch because this patient can die any time”*. (Nurse, Kenya)

A patient that needs close support is seen as being critically ill.

*“A critically ill patient is a patient who need close support from nurses or doctors”* (Doctor, Tanzania)

## Discussion

We have found that health workers understand the label ‘critical illness’ in diverse ways. The label ‘critical illness’ can be used for patients that (1) have a life threatening state; (2) are suffering from certain diagnoses; (3) are receiving care in certain locations; or (4) are in need of certain levels of care. Critical illness is a commonly used label in healthcare and yet appears to be understood differently by different health workers.

This variation in use is perhaps understandable given that there is not a universally agreed definition of critical illness in the literature. A recent scoping review has proposed the definition, “*a state of ill health with vital organ dysfunction, a high risk of imminent death if care is not provided and the potential for reversibility*” (23). The first understanding of the health workers in our study—that critical illness refers to patients in a life-threatening state—is closest to the proposed Kayambankadzanja definition, and in our opinion may be the most useful for health workers and health systems. A life-threatening state can be seen as one where there is a substantial risk of imminent death if action is not taken, and a label for situations where *risk* is the key concept would likely be beneficial. The label ‘critical illness’ can then capture all patients with a life-threatening condition, irrespective of location, type of disease or level of care a patient is receiving. If an understanding of the label ‘critical illness’ could be unified around the concept of risk and a life-threatening state, the identification and care of the group of patients sharing this attribute may be improved.

The other three could have shortcomings as understandings of the label ‘critical illness’ as they may lead to the inclusion of patients with diverse outcome risks. Understanding critical illness in terms of certain diagnoses such as eclampsia or head-injury may include stable patients with those conditions who do not require urgent intervention, and would miss patients in need of urgent care that are lacking such a diagnosis. Referring to critically ill patients as those being cared for in certain locations in hospitals, like in ICU or the emergency department, may exclude patients with a high risk of poor outcomes in other locations in the hospital. Additionally, patients in ICUs, in emergency departments or in other locations do not all have the same risks, prognosis or requirements for urgent care, so this understanding may direct care interventions inappropriately to patients who do not require them. This is a form of, “continuation bias”, which was identified as an important barrier to timely care of critically ill patients in Sri Lanka (24). Understanding ‘critical illness’ as referring to a situation where a certain level of advanced care such as mechanical ventilation is provided, will lead to the exclusion of patients who would benefit from such a label, but are either not in need of such advanced care, or who are cared for in settings where advanced care is not available (4).

The varied understandings of the label ‘critical illness’ may lead to challenges in care provision for critically ill patients. It could affect the quality of communication between health workers, as labels provide health workers with a framework which they use to organize and interpret clinical presentations, triage patients, support clinical decision making and treatment decisions, and provide information on prognosis (7, 9, 25). What will be said by one health worker around critical illness will not be what is understood by their colleague. Varied understandings may also impact the appropriation of resources for patient care (26).

Patient management can be affected by the varied understandings as labels create an assumption that all patients in label group are homogeneous (9), implying that the same care will be needed by all, and if the labelling has led to a group of patients with heterogeneous risks, prognoses and needs, this will not be appropriate. Labels determine health worker attitudes towards patients in terms of the required care (27, 28), a critical illness label will likely change health workers levels of attentiveness to patients—which patients require more attention and which are satisfied with less. Health workers are trained to call for help from other health workers or senior colleagues when a patient has needs that they cannot handle themselves. The timing of when to call for help will be affected by the labels given to the patient. Labels also influence decisions for referral which impact patient care, morbidity and mortality (29, 30). The decision to refer a patient to another care provider is based on the health workers’ understanding of the patient's needs, which in turn is built upon their use of labels such as critical illness. Variations in the understanding of critical illness can therefore lead to inappropriate decisions about care, attentiveness, the need to call for help and referral.

Among this study's strengths are a methodology that included gathering opinions from a range of cadres of health workers across specialties to capture broad perspectives. Existing established knowledge on labelling theory gave this study a sound reflection base to interpret the results and guide discussion. Limitations include the use of only five hospital sites and we cannot rule out selection bias in the processes of selecting participants. The lack of a generally agreed definition of critical illness precluded an assessment of health workers’ understanding against a standard.

Our findings stress the importance of developing a standard definition of critical illness and transmitting that knowledge to healthcare workers. The recently proposed definition by Kayambankadzanja and colleagues can provide a first-step towards this. We call for more studies from different settings to understand the impact of varied understandings of this key label on care provision and patient outcomes, and relatedly, for studies that would assess the impact of a standardized definition.

## Conclusion

There is a lack of unified understanding about the label critical illness among health workers in Tanzania and Kenya, potentially hampering communication and the selection of patients for urgent, life-saving care.

## Data Availability

The raw data supporting the conclusions of this article will be made available by the authors, without undue reservation.
